# Ethyl Formate Fumigation for Controlling Two Major Aphid Pests, *Aphis spiraecola* and *Aphis gossypii*, on Passion Fruit, from Cultivation to Post-Harvest Storage

**DOI:** 10.3390/insects15060386

**Published:** 2024-05-26

**Authors:** Jeong Oh Yang, Dongbin Kim, Young Su Lee, Ki-Jeong Hong, Byung-Ho Lee, Min-Goo Park

**Affiliations:** 1 Plant Quarantine Technology Center, Animal and Plant Quarantine Agency, Gimcheon 39660, Republic of Korea; joyang12@korea.kr; 2 Institute of Quality & Safety Evaluation of Agricultural Product, Kyungpook National University, Daegu 41566, Republic of Korea; kdb5924@naver.com (D.K.); byungholee@hotmail.com (B.-H.L.); 3 Gyeonggi-do Agricultural Research & Extension Services, Hwaseong 18388, Republic of Korea; yslee75@gg.go.kr; 4 Department of Plant Medicine, Sunchon University, 255 Jungang-ro, Suncheon 57922, Republic of Korea; curcul@scnu.ac.kr; 5 Department of Bioenvironmental Chemistry, Jeonbuk National University, Jeonju 54896, Republic of Korea

**Keywords:** ethyl formate, cultivation, passion fruit trees, post-harvest passion fruits, *Aphis spiraecola*, *Aphis gossypii*

## Abstract

**Simple Summary:**

In South Korea, the increase in growing tropical and subtropical crops due to climate change and consumer demand has brought along a challenge: invasive insect pests damaging these fruits. To address this issue, we explored using ethyl formate (EF) fumigation as a safer alternative to traditional methods. We focused on two types of aphids, common pests in the trade of fresh produce. We found that EF effectively controlled aphids across different stages of fruit cultivation and post-harvest storage, achieving 100% mortality without harming the plants. Importantly, EF levels rapidly decreased after fumigation, ensuring safety. These findings suggest that EF fumigation could be a valuable solution for protecting crops from pests in a changing climate. This research provides farmers and policymakers with a safer and more sustainable method for pest control in greenhouse and post-harvest storage, ensuring the availability and quality of tropical fruits for consumers while reducing environmental risks associated with traditional pesticides.

**Abstract:**

Tropical and subtropical crops are being increasingly cultivated in South Korea, leading to an increase in damage by exotic insect pests. Consequently, ethyl formate (EF) is currently being considered for quarantine and pre-shipment fumigation. In this study, we evaluated the effectiveness of EF fumigation for controlling *Aphis spiraecola* Patch and *Aphis gossypii* Glover, two representative quarantine pests on passion fruit (“Pink Bourbon”) during greenhouse cultivation and post-harvest storage. The efficacy of EF against both aphids in terms of the lethal concentration causing 50% mortality (LCt_50%_) and LCt_99%_ was 1.36–2.61 g h/m^3^ and 3.73–7.55 g h/m^3^ under greenhouse conditions (23 °C), and 1.37–2.02 g h/m^3^ and 3.80–14.59 g h/m^3^ post-harvest (5 °C), respectively. EF at 4 g/m^3^ for 4 h resulted in 100% mortality of *A. spiraecola*, which was more resistant to EF, without causing phytotoxic damage to the trees in a 340 m^3^ greenhouse. Post-harvest fruit fumigation at 10 g/m^3^ for 4 h in a mid-size (0.8 m^3^) fumigation chamber resulted in complete disinfection. Moreover, the EF level decreased below the EF threshold within 10 min after natural ventilation in the greenhouse. Therefore, our results suggest EF fumigation as an effective method for controlling *A*. *spiraecola* and *A*. *gossypii*.

## 1. Introduction

According to climate change predictions, the temperature is expected to rise by 4 °C by the end of the 21st century, which is projected to result in 17% of the Korean Peninsula transitioning into a subtropical climate zone [1]. One study identified 10 subtropical crops suitable for the changing Korean climate due to global warming, including passion fruit, mango, banana, dragon fruit, olive, papaya, guava, atemoya, coffee, and feijoa [2]. Facility horticulture has developed rapidly in Korea since the 1970s, and greenhouses have been established to grow various crops [3].

Passion fruit (*Passiflora edulis* Sims), originates from Brazil and stands as one of the most economically significant and extensively cultivated crops worldwide [4]. Brazil leads in production, contributing approximately 65% to the global output, with Colombia and Indonesia following suit as notable producers [5]. Passion fruit is preferred by farmers new to the market because of the low price of seedlings, low cost of initial investment, short time to harvest, and simple cultivation technology. In Korea, passion fruit orchards have increased from 43 in 2014 to 153 in 2016, and domestic consumption of tropical fruits is increasing [1]. While passion fruit is largely imported into Korea in a frozen state, there is an increasing demand for fresh fruits, considering that the import has risen by 225% from 990 tons in 2018 to 2228 tons in 2022 [6], making domestic cultivation a promising industry. When exporting fruits such as pears and apples outside Korea, there have been instances in which quarantine pests were found during inspections in those countries. Consequently, there has been a need for frequent fumigation with methyl bromide (MB) in these countries [7]. However, MB negatively impacts the value of the produce [8] and the health of the workers [9,10,11], making it necessary to replace MB with safer fumigants. Moreover, when passion fruits are exported, the pests in the fruits must be controlled before export.

*Aphis spiraecola* Patch and *Aphis gossypii* Glover are insect pests on citrus, fruit crops, and various perishable commodities, including passion fruit [12,13,14]. These aphids have also been reported to transmit a wide range of viruses, including cucumber mosaic, citrus tristeza, citrus tatter leaf, watermelon mosaic, and papaya ringspot viruses [12,13,15]. Specifically, aphids have been recognized to transmit virus-causing leaf mottling and ring spotting of passion fruit [13] and to spread cowpea aphid-borne mosaic virus in passion fruit orchards in Brazil [14]. Adults and nymphs are notorious for damaging crops by sucking host plant juices, which can lead to reduced productivity, and, in severe cases, the withering of crops. Despite the use of various types of insecticides to control aphids, resistance to insecticides has recently been reported in Korea [16]. As a result, controlling aphids in Korean cultivation areas has become increasingly difficult. The interception of the quarantine aphids, including those of the genera *Aphis*, *Myzus,* and *Ericaphis*, rose by 225% from 127 cases in 2020 to 249 cases in 2022 in Korea [6]. According to the increase, the risk of aphids against passion fruits is likely growing, although detection of the aphids has not been reported in the passion fruit orchard as it has only recently been introduced in Korea.

To combat these issues, the development of MB alternatives in the post-harvest storage condition and selection of effective methods for disinfection other than conventional pesticides, such as malathion, deltamethrin, and imidacloprid, is necessary in cultivation conditions.

Fumigation is a cost-effective and efficient method to disinfest insect pests [17]. In particular, EF has been reported as an effective pesticide for pests in a greenhouse that have developed resistance to other pesticides [18,19]. Several studies have explored the use of EF as a disinfectant on a range of commodities such as fruits, vegetables, nursery plants, and non-food commodities in the condition of post-harvest storage as quarantine purposes, and they have demonstrated its effectiveness [20,21,22,23,24,25,26]. In this study, we assessed the effectiveness of EF as a disinfectant for *A. spiraecola* and *A. gossypii*, two representative aphid species on tropical fruits, on passion fruit. Specially, our evaluation focused on three main objectives: (1) determining the efficacy of EF against *A. spiraecola* and *A. gossypii*, (2) conducting greenhouse EF fumigation on passion fruit trees at temperatures exceeding 23 °C and assessing phytotoxicity and worker safety, and (3) evaluating the efficacy of EF for post-harvest fruit storage at 5 °C and assessing sorption onto and phytotoxicity in passion fruit. 

## 2. Materials and Methods

### 2.1. Fumigants and Application

Safefume Inc. (Daegu, Republic of Korea) supplied 99% liquid EF (Fumate^TM^) and the necessary equipment for conducting fumigation experiments in both the laboratory and the greenhouse. In the laboratory experiments, which were conducted in a 0.8 m^3^ (0.8 m × 0.7 m × 1.4 m) fumigation chamber to simulate post-harvest passion fruit conditions, liquid ethyl formate was vaporized using an EF vaporizer and mixed with nitrogen carrier gas to form a non-flammable application. The greenhouse experiments were carried out in a 340 m^3^ (25 m × 4 m × 3.4 m) chamber used for cultivating mango trees, where an airless pump, provided by Safefume Inc., was used to spray liquid EF through a micro fine nozzle and vaporize it at natural temperature conditions inside the greenhouse.

### 2.2. Insects

*Aphis spiraecola* and *Aphis gossypii* were successively supplied in red pepper orchards, located in Gyeonggi-do, South Korea, and tested immediately. We placed the leaf cut into a round shape with the abaxial side facing upwards inside a Petri dish with a mesh lid and transferred the aphids onto the leaf from pepper. After fumigation, the mesh part of the Petri dish lid was covered with wet cotton to maintain moisture in the Petri dish until the end of the evaluation of mortality. For nymphs, the nymphal states (n3, n4) were used.

### 2.3. EF Concentration and Determination of the Ct (Concentration × Time) Product

During the fumigation process, EF concentration was analyzed using gas chromatography coupled with a flame ionization detector (GC-FID) following separation on an HP-5 Column (19091J-413; J&W Scientific, Folsom, CA, USA). The oven temperature was maintained at 150 °C, and the injector and detector temperatures were set at 240 °C. EF concentration was determined by comparing peak areas with external EF gas standards. Concentrations of EF were monitored at designated intervals of 0.5, 1.0, 2.0, and 4.0 h during exposure periods in either desiccators or the greenhouse. The *Ct* product was calculated as described by Ren et al. [27]:

(1) $$Ct=\sum \frac{\left(C_{i}+C_{i+1}\right)\left(t_{i+1}-t_{i}\right)}{2},$$

where *C* = concentration of the fumigant (mg/L), *t* = time of exposure (h), *i* = order of measurement, and *Ct* = the concentration × time product (g h/m^3^).

### 2.4. Efficacy of EF against A. spiraecola and A. gossypii in Laboratory Trials during Passion Fruit Tree Cultivation and Post-Harvest Passion Fruit Storage

The fumigation of *A. spiraecola* and *A. gossypii* using EF was conducted in glass desiccators (Duran^®^, 6.9 L) outfitted with a small fan placed at the bottom of each desiccator to ensure internal air circulation. Insect samples were introduced into insect breeding dishes (diameter: 4.5 cm) and positioned inside the desiccators. Once sealed with grease, liquid EF was injected into the desiccators using a gas-tight syringe (SGE Analytical Science, Trajan Scientific and Medical, Melbourne, Australia) according to a predetermined dosage calculated using the equation outlined by Ren et al. [25]. EF dosage ranged from 0.5 to 8 g/m^3^. The concentration of EF within the fumigation chamber was assessed by collecting gas samples at intervals of 0.1, 1.0, 2.0, and 4.0 h post-EF application. Gas samples were analyzed using a Shimadzu-GC 17A (Shimadzu, Kyoto, Japan) fitted with a flame ionization detector (FID) following separation on a DB5-MS Column (30 m × 0.25 mm i.d., 0.25 µm film thickness; J&W Scientific, Folsom, CA, USA). The oven temperature was maintained at 100 °C, with injector and detector temperatures set at 250 °C and 280 °C, respectively. Helium served as the carrier gas at a flow rate of 1.5 mL/min. EF concentration was determined by comparing peak areas with those of a series of external EF standards. Desiccators treated with EF were stored at 5 °C for post-harvest storage and at 23 °C for greenhouse conditions for 4 h. Following fumigation, *A. spiraecola* and *A. gossypii* were transferred to an insect rearing room under controlled conditions of 25 ± 2 °C and 75 ± 5% RH. Mortality of adults was assessed 72 h post-fumigation-treatment. Each experiment utilized more than 20 adults per replication, and all procedures were repeated three times, including a control group.

### 2.5. Post-Harvest Storage Condition of Passion Fruit at 5 °C

#### 2.5.1. Middle-Scale (0.8 m^3^) *A. spiraecola* Fumigation with Liquid EF

Middle-scale trials were conducted in a 0.8 m^3^ fumigation chamber to simulate post-harvest passion fruit conditions, using a 5% (*w*/*v*) loading ratio. For passion fruits, 5 and 10 g/m^3^ of EF was applied and held for 4 h at 5 °C. EF concentrations were monitored during fumigation as described in Section 2.4. Each fumigation chamber was fitted with a fan in the inner-top portion for air circulation. *A. spiraecola* in a breeding dish (diameter: 4.5 cm) was placed inside passion fruit boxes purchased from a local retailer. In this study, EF was administered at doses of 5 and 10 g/m^3^ using an EF vaporizer (SFM-1) within chambers maintained at 5 ± 1 °C, with samples undergoing fumigation for 4 h. Aphid mortality was assessed 72 h post-fumigation-treatment. Corrected mortalities were recalculated using Abbott’s formula [(Mort. of treatment − Mort. of control)/(1 − Mort. of control) × 100]. Untreated samples served as the control. A total of 653 adults and 630 nymphs of *A. spiraecola* were utilized, including controls. Sorption was calculated as the remaining ratio of concentration (*C*/*C*_0_), with C indicating the EF concentration measured at a specific time interval and *C_0_* representing the EF concentration at 0.1 h [20].

#### 2.5.2. Phytotoxic Assessments

The phytotoxic assessments on passion fruits were conducted in a 0.8 m^3^ fumigation chamber. Subsequently, 5% (*w*/*v*) loading ratios of passion fruit were selected. For passion fruits, 5 and 10 g/m^3^ of EF was applied and held for 4 h at 5 °C. The phytotoxic effects of passion fruit were assessed following 7 days of post-harvest storage conditions (5 ± 1 °C) regarding hardness, sugar content, color alteration, and weight loss subsequent to 4 h EF fumigation at concentrations of 5 and 10 g/m^3^. Hardness was gauged using a fruit firmness tester (53205 Digital fruit firmness tester, TR Turoni, Forli, Italy) equipped with an 8 mm steel plunger. Hardness measurements were conducted three times per fruit, with five fruits analyzed for each treatment. Soluble sugar content was determined using a portable refractometer (Hand refractometer ATC-1E, Atago Co., Ltd., Tokyo, Japan). The entire fruit was ground using a tissue grinder and filtered through a funnel covered with filter paper. A 0.5 mL portion of the filtered liquid was applied to the refractometer, and sugar content was assessed by observing the refractometer’s scope. Measurements were taken from five fruits per treatment. Color change was quantified using a colorimeter (TES 135A, Electrical & Electronic Corp., Taiwan, China), which expressed color through Hunter L*, a*, and b* values. Weight loss was calculated as the ratio between the weight before treatment and that after 7 days of storage.

### 2.6. Cultivation Conditions of Passion Fruit Tree at 23 °C

#### 2.6.1. Greenhouse Fumigation Using Liquid EF on *A. spiraecola*

Greenhouse fumigation of EF on passion fruit trees with 4 g/m^3^ for 4 h at 23 °C was conducted in a 340 m^3^ vinyl house located in Sancheong-gun, South Korea. At least 50 passion fruit individuals, planted in pots, were placed in the greenhouse. Since the crop is a vine plant, a string was attached to the top to enable it to climb up easily. Trials were conducted when the flowers had faded and the fruit had formed. 

*Aphis spiraecola* adults were introduced into insect breeding dishes (diameter: 4.5 cm) positioned at various locations within the vinyl house. Gas sampling lines were installed at three points (top, middle, and bottom) of the vinyl house to monitor EF concentration. Gas samples were collected at four intervals (0, 1, 2, and 4 h) and EF concentration was measured using GC-FID. In the greenhouse fumigation, EF was applied at a concentration of 4 g/m^3^ using a vaporizer for 4 h at 23 ± 2 °C. The EF concentration in the sampled gas was then analyzed using GC-FID. Following the 4 h fumigation period, the container was opened and ventilated for 1 h to reduce gas concentration, and the desorption rate was also measured using GC-FID. In total, 711 *A. spiraecola* adults were used for evaluation, including a control.

#### 2.6.2. Phytotoxic Assessments

In phytotoxic assessments, we evaluated the damage index using the following scale: 0 (no leaf damage), 1 (<5% of total leaves/plant dropped, browned, or shriveled), 2 (5–25% leaves affected), 3 (25–50% leaves affected), and 4 (>50% leaves affected). The chlorophyll levels of 10 leaves were assessed utilizing a chlorophyll meter (SPAD-502 Plus, Minolta, Tokyo, Japan). The color of 10 passion fruit tree leaves was measured using a colorimeter, and expressed in terms of Hunter L*, a*, b* values, with hue values calculated based on the following formula: hue = [L^2^ + a^2^ + b^2^]/2 (TES 135A, Electrical & Electronic Corp., Taipei, Taiwan, China). The assessments were conducted on passion trees and the mortality of adults was obtained using a microscope after fumigation for 3 days. 

#### 2.6.3. Worker Safety

Following the completion of greenhouse fumigation, the concentration within the vinyl house was periodically monitored for worker safety during the ventilation process using a portable gas analyzer (MiniRAE 3000, RAE Systems, San Jose, CA, USA).

### 2.7. Statistical Analysis

The toxicological dose response of EF on *A. spiraecola* was examined through Probit analysis. Quality parameter assessments including weight loss, firmness, sugar content, and surface color change were computed using Proc Univariate in SAS (ver. 9.4; SAS Institute Inc.). To assess the phytotoxic effects of EF fumigation on passion fruit, one-way analysis of variance (ANOVA) with Tukey’s studentized range (HSD) test at a significance level of *p* = 0.05 was conducted using SAS (ver. 9.4; SAS Institute Inc., Cary, NC, USA).

## 3. Results

### 3.1. Efficacy of 4 h EF Fumigation against A. spiraecola and A. gossypii during Cultivation and Post-Harvest Storage in Laboratory Trials

Under cultivation at 23 °C, the LCt_50%_ and LCt_99%_ of 4 h of EF fumigation for *A. spiraecola* were 1.36 and 4.45 g h/m^3^ for the nymph stage and 2.61 and 7.55 g h/m^3^ for the adult stage, as determined from the fitted slopes, 4.52 ± 0.4 and 5.04 ± 0.7. The LCt_50%_ and LCt_99%_ of 4 h of EF fumigation for *A. gossypii* were 2.49 and 4.42 g h/m^3^ for the nymph stage and 1.48 and 3.73 g h/m^3^ for the adult stage, as determined from the fitted slopes, 9.32 ± 1.8 and 5.78 ± 0.9.

In the post-harvest storage at 5 °C, the LCt_50%_ and LCt_99%_ of 4 h of EF fumigation for *A. spiraecola* were 1.62 and 14.59 g h/m^3^ for the nymph stage and 2.02 and 13.43 g h/m^3^ for the adult stage, as determined from the fitted slopes, 2.44 ± 0.2 and 2.82 ± 0.3 (Table 1). The LCt_50%_ and LCt_99%_ of 4 h of EF fumigation for *A. gossypii* were 1.71 and 4.44 g h/m^3^ for the nymph stage and 1.37 and 3.8 g h/m^3^ for the adult stage, as determined from the fitted slopes, 5.62 ± 1.0 and 5.26 ± 0.7. 

*Aphis spiraecola* was less sensitive to EF than *A. gossypii*. There was no significant difference in sensitivity to EF between nymphs and adults of *A. spiraecola*. The details are described in Table 1.

### 3.2. Effect of EF Fumigation during Post-Harvest Storage at 5 °C

#### 3.2.1. Mid-Scale (0.8 m^3^) EF Fumigation of *A. spiraecola*

**Figure 1:**
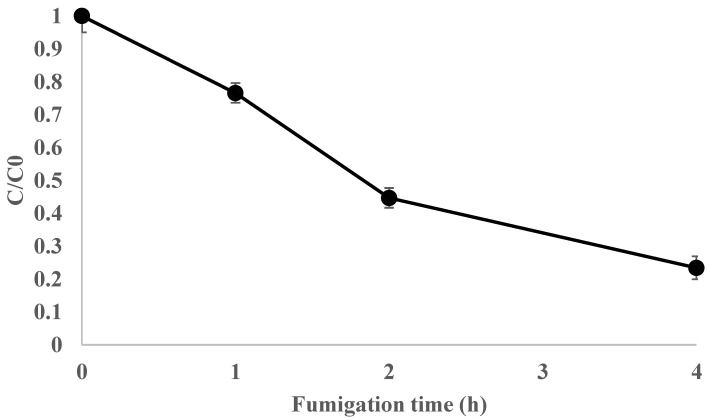
Ratio of the remaining ethyl formate concentration on passion fruits in post-harvest storage conditions (EF 10 g/m^3^ for 4 h at 5 ± 1 °C).

To assess the efficacy against *A. spiraecola,* the most EF-resistant species, during post-harvest storage, EF was applied to passion fruits at 5 or 10 g/m^3^, with a 5% (*w*/*v*) loading ratio, in a 0.8 m^3^ fumigation chamber. The mid-scale trial conditions and results are provided in Table 2. The *Ct* product for *A. spiraecola* at 5 g/m^3^ was 10.2 g h/m^3^, which was lower than the LCt_99%_. Not all 651 adults and 598 nymphs were killed in the 5 g/m^3^ trial. The *Ct* product for *A. spiraecola* at 10 g/m^3^ was higher than that for the LCt_99%_ (14.59 g h/m^3^). All 533 adults and 510 nymphs were completely eradicated at 10 g/m^3^. The mortality of untreated *A. spiraecola* was 0%. The ratio of remaining EF concentration in the fumigation chamber decreased during the 4 h fumigation period (Figure 1). Although the sorption of EF was approximately 75%, which was calculated from the remaining ratio of 25%, at the conclusion of the fumigation treatment, the *Ct* product was 19.8 ± 0.3 g h/m^3^ in the 10 g/m^3^ trial.

#### 3.2.2. Phytotoxic Effect of Post-Harvest EF Fumigation on Passion Fruits

**Figure 2:**
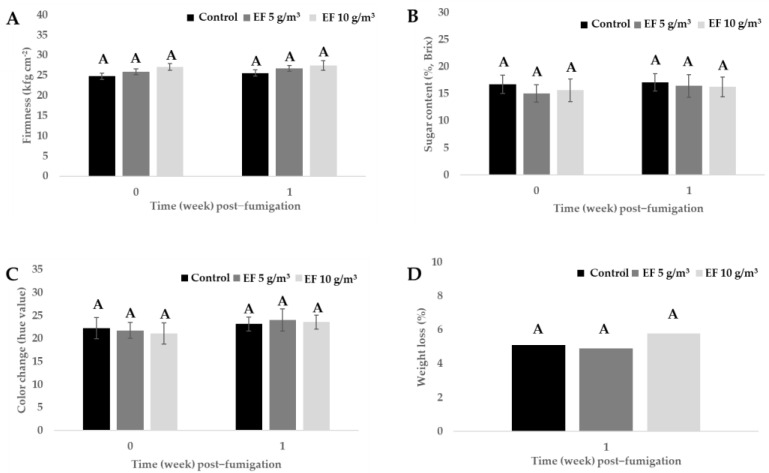
Phytotoxic assessments of passion fruits EF post−fumigation, treated with 5 and 10 g/m^3^ EF for 4 h in post−harvest storage conditions at 5 ± 1 °C temperature. (*Ct* products; EF 5 g/m^3^: 10.2 g h/m^3^, EF 10 g/m^3^: 19.8 g h/m^3^). The letter ‘A’ above each bar graph indicates no significant difference among the control, EF 5 g/m^3^, and EF 10g/m^3^ treatments. Fruit firmness (**A**), sugar content (**B**), color change (**C**), and weight loss (**D**).

In phytotoxicity assessments, fruit firmness, sugar content (%, brix), color change (hue value), and weight loss were measured 1 week post-fumigation (Figure 2A–D). The mean firmness values of untreated fruits and fruits treated with 5 or 10 g/m^3^ of EF 1 week post-fumigation were 25.6, 26.8, and 27.5 kgf/cm^2^, respectively. There was no significant difference in firmness according to an LSD test (0.38%, *p* = 0.05). The mean sugar content values of untreated fruits and fruits treated with 5 or 10 g/m^3^ of EF 1 week post-fumigation were 17.1%, 16.5%, and 16.3%, respectively, with no significant difference according to an LSD test (0.38%, *p* = 0.05). The mean color change values in untreated fruits and fruits treated with 5 or 10 g/m^3^ of EF 1 week post-fumigation were 23.2, 24.1, and 23.6, respectively, with no significant difference (0.38%, *p* = 0.05, LSD test). The mean weight loss values in untreated fruits and fruits treated with 5 or 10 g/m^3^ of EF 1 week post-fumigation were 5.1%, 4.9%, and 5.8%, respectively (0.38%, *p* = 0.05, LSD test). In conclusion, 4 h EF fumigation did not induce phytotoxic damage in terms of firmness, sugar content, color change, and weight loss during post-harvest storage.

### 3.3. Effect of EF Fumigation during Cultivation of Passion Fruit Tree at 23 °C

#### 3.3.1. Effect of Greenhouse Fumigation (340 m^3^) with Liquid EF on Passion Fruit Trees

Based on the laboratory trials on efficacy, 4 g/m^3^ of EF was applied to achieve an LCt_99%_ of 7.55 g h/m^3^ at 23 °C in *A. spiraecola* adults. The greenhouse fumigation trial conditions and results are provided in Table 3. Over 4 h of EF fumigation during cultivation at 23 °C, the concentration of EF systematically decreased because of EF sorption onto the passion fruit trees and soil, and other factors. The final EF concentration of EF at the end of fumigation was 35% of the initial dose. In greenhouse fumigation, the *Ct* products of EF were 8.9 ± 0.1, 7.9 ± 0.1, and 7.9 ± 0.1 g h/m^3^ for three greenhouse locations (top, middle, and bottom), respectively, which were higher than the LCt_99%_ of 7.55 g h/m^3^. All 591 *A. spiraecola* adults were disinfected at 4 g/m^3^.

#### 3.3.2. Phytotoxicity Assessments

In phytotoxicity assessments, chlorophyll content and color change under the cultivation conditions were investigated 1 week post-fumigation. The mean chlorophyll content values of untreated fruits and fruits treated with 4 g/m^3^ of EF 1 week post-fumigation were 20.1% and 19.8%, respectively, with no significant difference (0.38%, *p* = 0.05, LSD test). The mean color change values in untreated fruits and fruits treated with 4 g/m^3^ of EF 1 week post-fumigation were 28.7 and 30.3, respectively (0.38%, *p* = 0.05, LSD test). The decaying rates were 0% in both untreated fruits and fruits treated with 4 g/m^3^.

#### 3.3.3. Worker Safety

**Figure 3:**
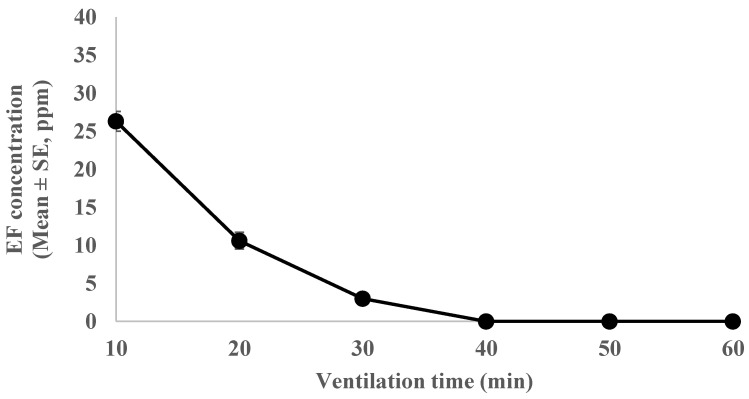
EF levels in the post-fumigation period after 4 h greenhouse fumigation in cultivation conditions (23 ± 1 °C, RH: 70–90%; current permeable level of EF is 100 ppm in Korea).

The EF concentration in the vinyl house rapidly decreased to below 100 ppm (TLV-TWA of EF) within 10 min of ventilation; however, it took more than 1 h to decrease the acute inhalation toxicity hazard in the workplace (Figure 3). 

## 4. Discussion

In the present study, EF treatment was effective in all growth stages of *A. spiraecola* and *A. gossypii* at 5 °C and 23 °C in laboratory trials. Adults and larvae were also successfully controlled in practical trials using a mid-scale 0.8 m^3^ fumigation chamber and a 340 m^3^ greenhouse. The optimal fumigation level was 10 g/m^3^ at 5 °C and 4 g/m^3^ at 23 °C for 4 h. This did not damage the fruits and trees and did not have a negative impact on worker safety as the EF concentration decreased to <100 ppm (TLV-TWA of EF) within 10 min of degassing.

Various pesticides, including bifenthrin, deltamethrin, imidacloprid, thiamethoxam, malathion, and acephate, have been used for aphid control [28,29,30]. However, controlling aphids can be challenging because of several factors, such as their very small body size, short generation time, genetic variability, and rapid development of pesticide resistance [31,32,33,34]. *Aphis spiraecola* and *A. gossipy* have a wide range of host plants [15,35], including citrus, mango, passion fruit, strawberry, and even flowers such as rose and hibiscus. Their phloem feeding behavior and habit of hiding on the abaxial surfaces of leaves or within curled-up plant parts not only make aphids challenging to target with contact pesticides, but also reduce the effectiveness by decreasing the contact area, leading to an increase in the amount of pesticide usage [31]. Gas-type EF fumigation can increase the effectiveness because the gas penetrates the space in the greenhouse, reaching the aphids that can otherwise quickly escape.

In a previous study, *Myzus persicae* Sulzer were controlled effectively by EF treatment [36]. At 5 °C, the LCt_99%_ values for *M. persicae* were 6.95 and 7.66 g h/m^3^ for the nymph and adult stages. At 23 °C, they were 4.69 and 4.45 g h/m^3^ for the nymph and adult stages, respectively. In turnip aphids (*Lipaphis erysimi* Kaltenbach) treated with EF at a *Ct* product of 4 g h/m^3^ or less for 2 h at 5 °C or 20 °C, both nymphs and adults showed 100% mortality (unpublished data BHL). Consistent with these findings, in our study, the LCt_99%_ values were 4.45 and 7.55 g h/m^3^ at 23 °C for nymphs and adults of *A. spiraecola*, and 4.42 and 3.73 g h/m^3^ for *A. gossypii*.

The potential harm to plants from EF has been previously assessed across a range of plants grown in nurseries [20,25], as well as in fruits [21,26,37,38] and vegetables [39]. These studies indicated that the extent of the harmful effect depended on factors such as the treatment amount used and the plant species and variety. When applied to fruits such as oranges, bananas, grapes, and persimmons, EF demonstrated no negative impact on overall fruit quality [21,26,37,38]. In nursery plants, the observed levels of EF-induced plant damage varied, ranging from absence of harm (for instance, in *Ficus benghalensis* Banyan and *Cymbidium goeringii* Reichenbach) to significant harm (as observed in *Anthurium andraeanum* Linden and *Agave attenuata* Salm-Dyck) [20,25]. In this study, there were no significant changes in fruit firmness, sugar content, color change, and weight loss 1 week after the EF treatment at 5 or 10 g/m^3^ at 5 ± 1 °C (Figure 2). Further, the chlorophyll content and color of passion fruit trees did not change in the 340 m^3^ greenhouse after EF treatment 4 g/m^3^ at 23 ± 1 °C. It is important to note that passion fruits are used in traditional medicine as sedatives and anxiolytics and reportedly have various biological activities, such as antioxidant, anti-inflammatory, antibacterial, gastroprotective, antidiarrheal, analgesic, antiproliferative, and anti-diabetic effects, owing to bioactive compounds [40,41,42,43,44,45,46]. Passion fruits are also a good source of micronutrients as they contain high levels of vitamin C, potassium, copper, magnesium, zinc, and iron [47]. Therefore, further research will be required to examine changes in these bioactive compounds in passion fruits before and after EF fumigation.

However, the limitation of our study lies in our assessment of only two aphids. It remains unclear whether EF can effectively eradicate other exotic aphid species. Therefore, further research is needed, including assessment of the effectiveness of EF against different aphid species and re-assessment of potential harm to plants if more EF-tolerant species exist, as well as confirmatory trials using passion fruit or passion fruit trees infested by exotic aphids or similarly resistant replacements.

In conclusion, exposing greenhouse passion fruit trees to EF fumigation at a concentration of 4 g/m^3^ for 4 h at 23 °C in a cultivation scenario resulted in 100% mortality of *A. spiraecola* and *A. gossypii*. This fumigation treatment did not cause any harmful effects on the plants at levels higher than the EF LCt_99%_ value of 7.55 g h/m^3^ at 23 °C for 4 h, and EF concentrations decreased to below 100 ppm of TLV after 10 min of ventilation, causing no harm to workers. Fresh passion fruits subjected to treatment with EF at 10 g/m^3^ for 4 h at 5 °C in a post-harvest scenario did not exhibit any harm in terms of quality at levels above the EF LCt_99%_ value (14.59 g h/m^3^ for 4 h at 5 °C). We conclude that EF fumigation, which is currently being implemented as a substitute for MB in quarantine and pre-shipment procedures in Korea, shows potential as an effective method for controlling *A. spiraecola* and *A. gossypii*. Moreover, it could prove beneficial in addressing unforeseen infestations of exotic pests from cultivation in the greenhouse to storage of the fruits after harvesting, all while preserving the quality of the produce.

## Figures and Tables

**Table 1:** Efficacy of 4 h ethyl formate fumigation to aphids in greenhouse cultivation conditions at 23 °C and 5 °C.

Temp. (°C)	Target Insect	Developmental Stage	Number of Individuals Treated	LCt_50%_^a^ (95% CI, g h/m^3^)	LCt_99%_^a^ (95% CI, g h/m^3^)	Slope ± SE ^b^	*df* ^c^	*X* ^2 d^
23	*Aphis spiraecola*	Nymph	240	1.36 (1.24–1.48)	4.45 (3.70–5.74)	4.52 ± 0.4	6	10.26
		Adult	265	2.61 (2.19–3.00)	7.55 (5.97–11.33)	5.04 ± 0.7	7	40.25
	*Aphis gossypii*	Nymph	540	2.49 (2.12–2.77)	4.42 (3.77–6.29)	9.32 ± 1.8	15	4.88
		Adult	540	1.48 (0.85–1.95)	3.73 (3.18–4.42)	5.78 ± 0.9	15	2.07
5	*Aphis spiraecola*	Nymph	600	1.62 (1.37–1.86)	14.59 (11.11–21.07)	2.44 ± 0.2	25	37.36
		Adult	540	2.02 (1.62–2.46)	13.43 (9.64–21.85)	2.82 ± 0.3	22	47.39
	*Aphis gossypii*	Nymph	480	1.71 (1.12–2.04)	4.44 (3.66–6.79)	5.62 ± 1.0	11	4.29
		Adult	480	1.37 (1.00–1.64)	3.80 (3.24–5.07)	5.26 ± 0.7	11	2.33

^a^ LCt_50%_ and LCt_99%_ with 95% CI indicate the time at which 50% and 99% lethality of the aphids are achieved with a 95% confidence interval, respectively. ^b^ Slope indicates the relationship between the time at a certain temperature and the lethality of the aphids. SE, standard error. ^c^ Degrees of freedom. ^d^ *X*^2^ based on pooling of data with low expectation.

**Table 2:** Mortality of *Aphis spiraecola* adults and nymphs according to ethyl formate concentration and *Ct* products by location after 4 h ethyl formate middle-scale fumigation (0.8 m^3^) in post-harvest storage conditions (5 ± 1 °C, RH: 70–90%).

Applied Dose (g/m^3^)	Exposure Time (h)	EF Concentration (Mean ± SE, g/m^3^)			Mortality ^1^ (Mean ± SE, %)	
		Top	Middle	Bottom	Adults	Nymphs
5	0.1	4.7 ± 0.1	4.2 ± 0.1	4.4 ± 0.1	92.8 ± 1.8	87.3 ± 4.0
	1.0	3.6 ± 0.1	3.4 ± 0.2	3.9 ± 0.2		
	2.0	2.1 ± 0.1	2.0 ± 0.1	2.4 ± 0.1		
	4.0	1.3 ± 0.1	1.2 ± 0.2	1.4 ± 0.1		
	*Ct* products (Mean ± SE, g h/m^3^)	10.2 ± 0.1	9.5 ± 0.1	10.9 ± 0.1		
10	0.1	9.4 ± 0.1	9.1 ± 0.1	9.6 ± 0.1	100 ± 0.0	100 ± 0.0
	1.0	7.2 ± 0.0	7.0 ± 0.1	7.4 ± 0.0		
	2.0	4.2 ± 0.1	4.1 ± 0.1	4.4 ± 0.1		
	4.0	2.2 ± 0.1	2.1 ± 0.1	2.2 ± 0.1		
	*Ct* products (Mean ± SE, g h/m^3^)	19.8 ± 0.1	19.2 ± 0.1	20.4 ± 0.1		

^1^ Corrected mortalities were recalculated based on Abbott’s formula [Mort. of treatment − Mort. of control)/(1 − Mort. of control) × 100]. SE, standard error.

**Table 3:** Mortality of *Aphis spiraecola* adults according to ethyl formate concentration and *Ct* products by location after 4 h ethyl formate greenhouse fumigation (340 m^3^) in cultivation conditions (23 ± 1 °C, RH: 70–90%).

Applied Dose (g/m^3^)	Exposure Time (h)	EF Concentration (Mean ± SE, g/m^3^)			Mortality ^1^ (Mean ± SE, %)
		Top	Middle	Bottom	
4	0.1	3.6 ± 0.1	3.4 ± 0.0	3.5 ± 0.1	100 ± 0.0
	1.0	3.0 ± 0.0	3.0 ± 0.1	3.0 ± 0.0	
	2.0	2.4 ± 0.0	1.6 ± 0.1	1.5 ± 0.0	
	4.0	1.0 ± 0.1	1.0 ± 0.1	1.1 ± 0.0	
	*Ct* products (Mean ± SE, g h/m^3^)	8.9 ± 0.1	7.9 ± 0.1	7.9 ± 0.1	

^1^ The corrected mortalities were recalculated based on Abbott’s formula [Mort. of treatment − Mort. of control)/(1 − Mort. of control) × 100]. SE, standard error.

## Data Availability

All the data supporting the findings of this study are available from the corresponding authors upon reasonable request.
